# Persistent sarcopenia after flare remission in experimental RA responds to dipyridamole and is associated with restoration of adenosine and A2BR signaling

**DOI:** 10.3389/fphar.2026.1949677

**Published:** 2026-10-20

**Authors:** Miguel Marco-Bonilla, Maria Fresnadillo, Fernando Huete-Toral, Gonzalo Carracedo, Gabriel Herrero-Beaumont, Raquel Largo, Aránzazu Mediero

**Affiliations:** 1 Joint and Bone Research Unit, FIIS-Fundación Jiménez Díaz UAM, Madrid, Spain; 2 PhD Program in Medicine and Surgery, Universidad Autónoma, Madrid, Spain; 3 Ocupharm Group Research, Faculty of Optic and Optometry, University Complutense of Madrid, Madrid, Spain; 4 Department of Optometry and Vision, Faculty of Optic and Optometry, University Complutense of Madrid, Madrid, Spain

**Keywords:** adenosine, inflammatory remission, purinergic system, rheumatoid arthritis, sarcopenia

## Abstract

**Introduction:**

Rheumatoid arthritis (RA) is associated with systemic inflammation and sarcopenia, which often persist despite successful control of joint swelling and the mechanisms sustaining it remain unclear.

**Methods:**

Using the K/BxN serum-transfer model, we evaluated muscle structure, metabolism, and function after inflammatory remission. Dipyridamole was administered after remission of joint swelling to assess whether enhancement of adenosine and adenosine receptor signaling restore muscle homeostasis. Histological, molecular, metabolic, and functional analyses were performed.

**Results:**

RA mice exhibited persistent loss of muscle mass, fiber atrophy, fibrosis, white adipose tissue expansion, and impaired strength and endurance despite normalization of joint swelling. RA muscle showed sustained myostatin (MSTN) elevation, Akt/mTOR suppression, impaired regeneration, senescence markers, and disrupted purinergic signaling characterized by reduced adenosine levels, diminished adenosine A2B receptor (A2BR) expression, and decreased AMP-activated protein kinase (AMPK) activation. Dipyridamole administered after remission of joint swelling restored muscle adenosine availability, reactivated A2BR–AMPK signaling, suppressed MSTN, stimulated insulin-like growth factor 1 receptor (IGF1R)-dependent anabolic pathways, reduced fibrosis, and improved muscle function and fiber composition.

**Conclusion:**

RA induces persistent sarcopenia that remains after inflammation resolution. Post-remission dipyridamole reverses this phenotype in association with restoration of adenosine signaling, increased A2BR expression, AMPK activation, and enhanced anabolic pathways, highlighting purinergic modulation as a promising therapeutic strategy to improve muscle integrity and function in RA remission.

## 1 Introduction

Rheumatoid arthritis (RA) is a chronic autoimmune disease which affects joints but also other tissues due to systemic inflammation. It alters body composition by reducing corporal weight and muscle mass in a comorbidity called sarcopenia. Its prevalence ranges from 20%–44%, reflecting substantial variability across studies due to the lack of standardized diagnostic criteria (Bennett et al., 2023).

Beyond its effects on physical function, rheumatoid sarcopenia has emerged as a clinically relevant comorbidity associated with disability, reduced quality of life, frailty and adverse metabolic outcomes. Increasing evidence indicates that alterations in muscle mass and strength may occur early during disease progression and can persist despite adequate control of joint inflammation, suggesting that muscle tissue may undergo long-lasting pathological changes independent of active synovitis (Giles et al., 2008; Costamagna et al., 2015; Moschou et al., 2023).

Conventional treatments such as methotrexate (MTX) and biological DMARDs (disease-modifying antirheumatic drugs) effectively control joint and systemic inflammation but frequently fail to restore muscle homeostasis, leaving a persistent sarcopenic state with unclear clinical features and therapeutic management (Hein et al., 2022; Hasegawa et al., 2023; Kulyk and Dzhus, 2025). Consequently, identifying the mechanisms responsible for persistent muscle impairment is essential for the development of therapies targeting rheumatoid sarcopenia beyond inflammatory control.

Purinergic system, involving adenosine and adenosine triphosphate (ATP), regulates inflammation. Adenosine receptors A2A (A2AR) and A2B (A2BR) regulate inflammation, with A2BR supporting muscle maintenance and strength (Ohta and Sitkovsky, 2014; Gnad et al., 2020; Vincenzi et al., 2023).

Among potential therapeutic approaches, modulation of adenosine signaling is particularly attractive because it influences both inflammatory and metabolic pathways. Dipyridamole, a phosphodiesterase (PDE)3 inhibitor and nucleoside transport blocker, is an FDA and EMA approved drug currently used as an antiplatelet and vasodilatory agent for thromboembolic prevention, with an established safety profile and extensive clinical experience. Drug repurposing strategies based on approved compounds may accelerate clinical translation while reducing development costs and safety concerns compared with *de novo* drug discovery (Allahham et al., 2022; Pinzi et al., 2024).

Dipyridamole increases extracellular adenosine, enhancing A2AR and A2BR expression, counteracting both systemic and muscle inflammation in experimental RA (Marco-Bonilla et al., 2025). Besides reducing inflammation, dipyridamole restored muscle mass through A2BR-dependent anabolic signaling (Marco-Bonilla et al., 2025). However, it is not described whether RA-associated sarcopenia persists once inflammation has resolved (sarcopenic imprint), or whether dipyridamole treatment initiated after inflammatory remission can restore muscle homeostasis. Therefore, this study aims to investigate whether experimental RA induces a persistent sarcopenic imprint following spontaneous clinical remission and to evaluate whether post-remission dipyridamole administration reverses this imprint through purinergic signaling.

## 2 Materials and methods

All the reagents and resources used are defined in Supplementary Table S1.

### 2.1 K/BxN serum transfer-induced RA model

Animal management and experimentation complied with the Spanish Regulations and the EU Guidelines for the Care and Use of Laboratory Animals and were approved by the Institutional Ethics and Welfare Committee of the Jiménez Díaz Foundation University Hospital and Research Institute (PROEX 19/019 and PROEX 019.6/24).

K/BxN mice were generated by crossing KRN mice with NOD/Lt mice. Serum was collected from adult arthritic K/BxN mice and pooled for experimental use. Twelve-week-old, age-matched male C57BL/6J mice (Charles River Laboratories, Barcelona, Spain) were randomly assigned to three experimental groups employing research randomizer free software: (1) Sham (healthy): No serum transfer (N = 7); (2) RA (Arthritic control): Mice received 100 μL of pooled serum via intraperitoneal (IP) injection on days 0 and 2 to induce arthritis and vehicle (5% ethanol in PBS) IP daily starting on day 28 (N = 7); (3) RA + Dipy: Arthritic mice treated with dipyridamole at 25 mg/kg/day diluted in vehicle IP, administered daily starting on day 28, along with serum transfer (100 μL IP on days 0 and 2) (N = 7).

Previously, it had been identified that the inflammatory autoresolution and persistent muscle decline occurred on day 28 in K/BxN RA mice model (Marco-Bonilla et al., 2025). RA progression was evaluated daily and the severity of RA in each paw was assessed using a 4-point scale as previously described (Marco-Bonilla et al., 2025). Mice were weighed weekly and sacrificed on day 45 after serum injection. Representative paw photographs were taken on days 15 and 28. Gastrocnemius (GA) was collected for protein and RNA extraction or nucleotide analysis via HPLC. Tibialis Anterior (TA) was included in OCT for freeze histology. GA, TA, white adipose tissue (WAT) and brown adipose tissue (BAT) mass were correlated to mice body weight.

### 2.2 Motor activity tests

Ambulatory activity, two-limb hanging, forelimb grip strength, and rotarod tests were conducted before serum transfer and on weekly bases for six weeks, as we previously described (Marco-Bonilla et al., 2025). Briefly, ambulatory activity was assessed by recording 6 min of free movement using a mobile device, with mice placed in individual chambers, and subsequently measuring the distance travelled. The two-limb-hanging test was performed following the protocol described by Aartsma-Rus and van Putten (Aartsma-Rus and Van Putten, 2014). Forelimb grip strength test was measured three times, and the average peak force of the forelimbs was calculated. Finally, rotarod test required a previous training before baseline measurement. After training, mice performed three trials on an accelerating rotarod (4–40rpm over five minutes), and each trial ended when the mouse fell from the rod. All results are expressed relative to baseline.

### 2.3 Histological analysis of TA

Histological analyses (H&E, ATPase, Picrosirius Red, and IgG/laminin staining) were performed as previously described (Marco-Bonilla et al., 2025) from 10 μm frozen sections brought to room temperature. Briefly, for H&E staining, sections were fixed in methanol for 30 min and stained with H&E. To analyze cross-sectional area (CSA), 3 representative 20X images per mice were processed with ImageJ software. After calibration with a scale bar, complete muscle fibers were manually outlined. CSA was determined using the Analyze Particles function (size threshold 20 μm^2^ to infinity), and individual fiber areas were exported for analysis.

In the case of ATPase, sections were immersed in a solution of 0.1M glycine/NaCl buffer ph 9.4, with 0.75M CaCl_2_ and 5 mg ATP for 45 min. After a rinse in water, sections were placed in 2% cobalt chloride solution, subsequently treated with 1:10 ammonium chloride to reveal type I and II fibers (light and dark respectively). Sections were finally dehydrated and mounted with DPX mounting medium. Then, 3 representative 20X images per mice were analyzed with ImageJ to evaluate the percentage of dark and light fibers. Both fiber types were counted manually using the Cell Counter plugin. For each animal, the mean number of dark and light fibers was calculated and expressed as a percentage of the total fibers counted. These data were used for final analysis.

Picrosirius red staining started with 30 min fixation in 4% paraformaldehyde (PFA). Sections were then incubated for 90 min in 0.1% Sirius Red solution in aqueous saturated picric acid. Following this, sections were immersed in 0.1M HCl solution for 1 min and then washed two times with water. Finally, they were dehydrated and mounted with DPX. 3 representative 20X images were analyzed with ImageJ software. Picrosirius staining was quantified using the green channel after RGB color separation. A uniform threshold was applied to all images. Collagen-positive area, and mean intensity were measured for statistical analysis.

For necrosis staining with IgG, sections were first fixed in PFA and washed before blocking with 5% bovine serum albumin (BSA) in phosphate-buffered saline (PBS) for 1 h. Then, sections were incubated overnight with laminin antibody in a humid chamber. After that, sections were washed and incubated for 1 h with secondary Alexa Fluor 594 red-fluorescent antibody to rabbit IgG and secondary Alexa Fluor 488 green-fluorescent antibody to mouse IgG. 3 representative 20X images were taken to determine necrosis in TA using ImageJ software. Total fibers and fibers with intracellular IgG staining were manually counted using the Cell Counter plugin. Necrosis was expressed as a percentage of positive IgG fibers relative to the total number of fibers analyzed.

### 2.4 Western blot

Proteins were extracted using RIPA buffer supplemented with protease and phosphatase inhibitors from GA pulverized tissue. Protein concentrations were determined by BCA assay. 40 μg of protein were separated by 6%–10% SDS-PAGE gels and transferred to nitrocellulose membranes. They were blocked with 3% BSA in TBS-T and incubated overnight at 4 °C with primary antibody. After washing, membranes were incubated with a secondary HRP-conjugated antibody for 1 h. Protein bands were visualized using an HRP and an Amersham Imager 600. Tubulin was used as a loading control. Quantity One was used to determine band´s intensity and protein levels were then normalized to tubulin levels and expressed as a percentage relative to sham mice. Details are described previously (Marco-Bonilla et al., 2025). Antibodies employed are listed in Supplementary Table S1.

### 2.5 RNA isolation and RT-PCR

Total RNA was extracted from pulverized GA tissue using TRIzol reagent following manufacturer’s instructions and quantified using a Nanodrop. 1 μg of RNA was reverse-transcribed into cDNA using the High-Capacity cDNA Reverse Transcription Kit. Gene expression was analyzed by quantitative real-time PCR (RT-qPCR) using PowerUp SYBR Green Master Mix on a StepOnePlus Real-Time PCR System. Relative gene expression levels were calculated using the 2^−ΔΔCt^ method. Primer sequences are listed in Supplementary Table S2.

### 2.6 Determination of nucleotide concentration via HPLC

GA tissue was homogenized in a lysis buffer containing protease and phosphatase inhibitors and erythro-9-(2-hydroxy-3-nonyl) adenine (EHNA) to prevent adenosine degradation. Samples were sonicated, deproteinized with perchloric acid and potassium hydroxide, and centrifuged. Supernatants were collected and stored at −80 °C until analysis. Concentrations of adenosine, inosine, AMP, ADP, and ATP were determined by high-performance liquid chromatography (HPLC) using a reverse-phase column and UV detection at 254 nm. Compounds were identified and quantified by comparison with authentic standards based on retention times and calibration curves as previously described (Marco-Bonilla et al., 2025).

### 2.7 Statistical analysis

Sample size was determined *a priori* as part of the ethical approval process using the AEEC Animal Experimentation Sample Size Calculator, based on preliminary data obtained from our previous study in the same K/BxN serum-transfer RA model. In that study, RA mice exhibited a persistent reduction in body weight at day 28, despite resolution of joint swelling. Since body weight loss in this model is closely associated with muscle wasting and sarcopenia, this variable was selected as the primary endpoint for sample size estimation and for treatment initiation in the present study. Based on the percentage change in body weight from baseline at day 28 (healthy mice: 96.94% ± 4.37%; RA mice: 86.54% ± 5.95%), a sample size of 7 animals per group was estimated to provide 95% power at an α level of 0.05. This estimation was independently confirmed using G*Power 3.1.9.7, yielding the same required sample size (n = 7/group; Cohen’s d = 1.99; actual power = 96.9%).

Body weight recovery following dipyridamole treatment was defined *a priori* as the primary outcome measure. All other endpoints were considered secondary or exploratory outcomes. Data normality was assessed using the Kolmogorov-Smirnov test. Longitudinal measurements, including paw thickness, clinical score, body weight, and motor function assessments, were analyzed using a two-way repeated-measures ANOVA with treatment and time as factors, followed by Tukey’s multiple-comparisons test. All other normally distributed datasets were analyzed using one-way ANOVA followed by Tukey’s multiple-comparisons test. Non-normally distributed data were analyzed using the Kruskal-Wallis test. Outliers were identified using the ROUT method (Q = 1%). Four observations were excluded from the final analyses: one Sham sample in the CSA analysis, one RA + Dipy sample in the Pax7 analysis, one RA + Dipy sample in the AMP/ATP ratio analysis, and one RA sample in the Murf-1 analysis. The corresponding final sample size for these specific analyses was therefore n = 6 in the affected group. Reanalysis including these observations did not alter the overall interpretation of the results. Statistical analyses and graphical representations were performed using GraphPad Prism version 10. A p-value <0.05 was considered statistically significant. Data is presented as mean ± SEM, with individual data points displayed whenever possible.

## 3 Results

### 3.1 Dipyridamole counteracts the sarcopenic phenotype in RA mice after inflammatory remission

We previously showed that 28 days after K/BxN serum induction in mice, systemic and muscular inflammatory cytokines and joint swelling were similar to healthy controls, with persistent muscle atrophy (Marco-Bonilla et al., 2025). Thus, dipyridamole was administered at this time. As expected, RA mice developed paw arthritis, peaking at day 15 and resolving by day 28 (Figure 1A). Dipyridamole increased body weight by week 2 of treatment (week 6 post-induction), although not to Sham levels (Figure 1B). It also reversed persistent macroscopic muscle loss (Figure 1C).

**FIGURE 1:**
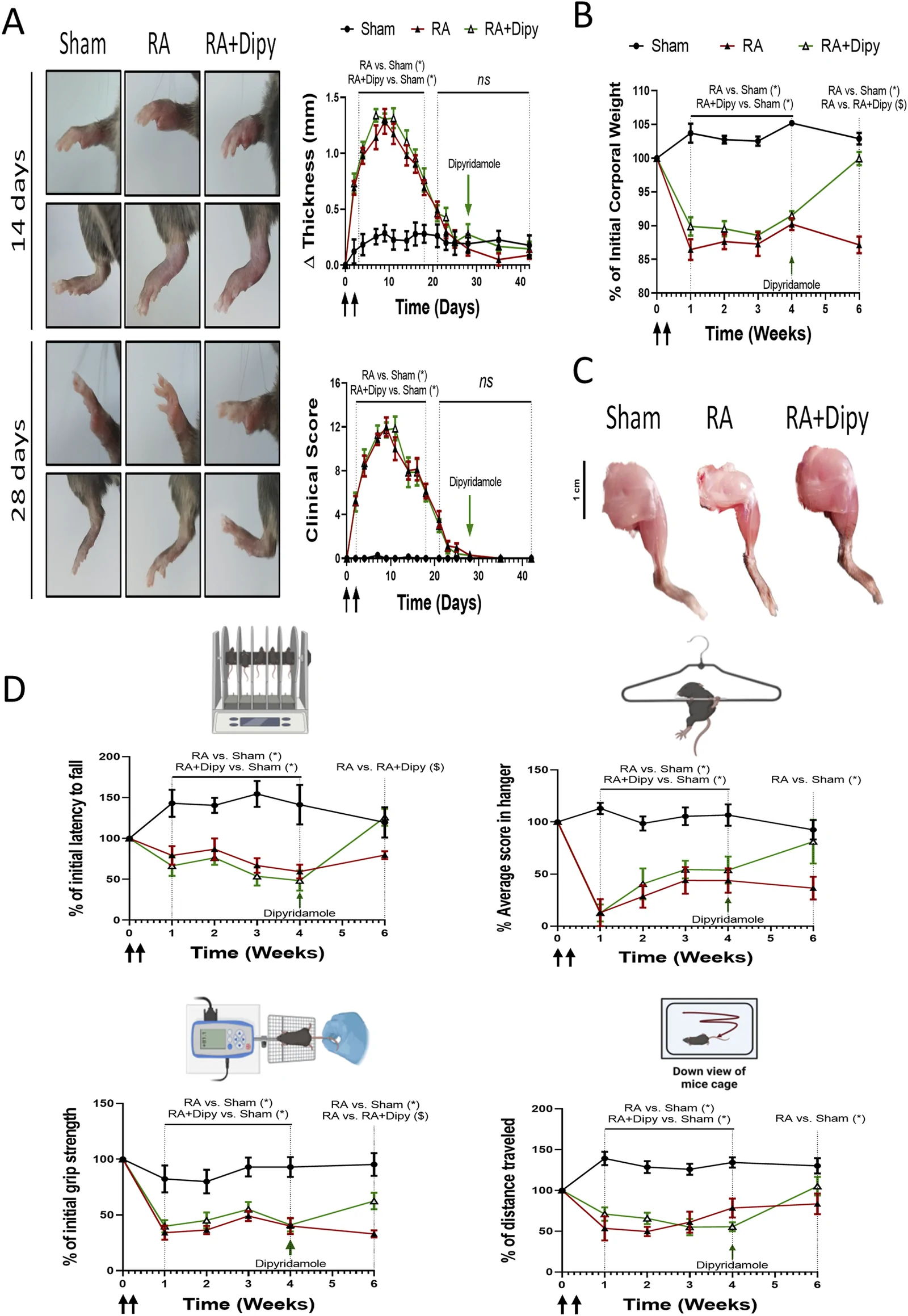
Dipyridamole counteracts the sarcopenic phenotype in RA mice after remission. **(A)** Macroscopic photography’s, thickness and clinical score analysis of hind and fore limbs of mice. **(B)** Body weight changes from week 0–6 after K/BxN serum injection. **(C)** Representative images of hind limb muscles at day of sacrifice. **(D)** Motor activity tests of two limb hanging test, rotarod, forelimb grip strength test and ambulatory activity represented as difference between the values obtained at week 0 before RA induction. Black arrows indicates the days of K/BxN serum administration. Green arrows represent the starting day of therapeutic dipyridamole administration. Differences are represented as: * Sham vs other groups; $ RA vs. RA + Dipy. Single symbol represents p < 0.05, double symbol represents p < 0.01 and triple symbol represents p < 0.005. Results are presented as mean ± SEM. N = 7 per group. RA: Rheumatoid Arthritis; RA + Dipy: Therapeutic treatment with dipyridamole from week 4 after RA induction.

Up to week 4, RA mice showed reduced physical activity in all tests. By week 6, motor coordination recovered in the rotarod, further improved by dipyridamole (Figure 1D). However, grip strength, ambulation, and hanging endurance remained reduced. Dipyridamole restored these parameters to Sham levels by week 6 (Figure 1D).

Previously, we reported increased WAT and decreased BAT, GA, and TA at day 15 (the peak of inflammation) (Marco-Bonilla et al., 2025). After remission, increased WAT and reduced TA and GA mass persisted (Table 1). In contrast, BAT mass was restored upon remission. Treatment with dipyridamole counteracted these effects by promoting BAT, GA, and TA mass while reducing WAT mass in RA mice (Table 1).

**TABLE 1:** Total and relative tissue weights collected at the time of sacrifice. Differences are represented as.

Study groups	​	Absolute weight (g)	Relative weight (% of body weight)
Sham	Body weight	29.13 ± 0.47	/
	Gastronemius	0.036 ± 0.002	0.12 ± 0.01
	Tibialis	0.018 ± 0.0006	0.06 ± 0.002
	WAT	0.026 ± 0.003	0.09 ± 0.009
	BAT	0.015 ± 0.0008	0.05 ± 0.003
RA	Body weight	24.9 ± 0.25	/
	Gastronemius	0.024 ± 0.0004	0.96 ± 0.001**
	Tibialis	0.01 ± 0.0004	0.04 ± 0.001***
	WAT	0.032 ± 0.004	0.13 ± 0.016
	BAT	0.01 ± 0.0008	0.04 ± 0.003
RA+Dipy	Body weight	29.14 ± 0.25	/
	Gastronemius	0.033 ± 0.0007	0.11 ± 0.002 $
	Tibialis	0.015 ± 0.0006	0.05 ± 0.002*/$$
	WAT	0.018 ± 0.001	0.06 ± 0.003 $$$
	BAT	0.016 ± 0.001	0.05 ± 0.003 $

* Sham vs other groups; $ RA vs. RA + Dipy. Single symbol represents p < 0.05, double symbol represents p < 0.01 and triple symbol represents p < 0.005. Results are presented as mean ± SEM. N = 7 per group. RA: Rheumatoid Arthritis; RA + Dipy: Therapeutic treatment with dipyridamole from week 4 after RA induction; GA: Gastrocnemius; TA: Tibialis Anterior; WAT: White Adipose Tissue; BAT: Brown Adipose Tissue.

All these results demonstrate that our mice model replicates human sarcopenia, with loss of body weight and muscle mass.

### 3.2 Dipyridamole reverses muscle fiber atrophy, fibrosis, and myogenic dysregulation in RA mice after inflammatory remission

Reduced TA mass in RA mice was associated with fiber atrophy (Figure 2A). Type II muscle fibers predominate in RA mice, unlike type I in Sham mice (Figure 2B). Additionally, fibrosis was evident in the TA muscle of RA mice (Figure 2C). Dipyridamole attenuated these alterations, restoring type I fibers and limiting collagen deposition to the sarcolemma (Figures 2A–C).

**FIGURE 2:**
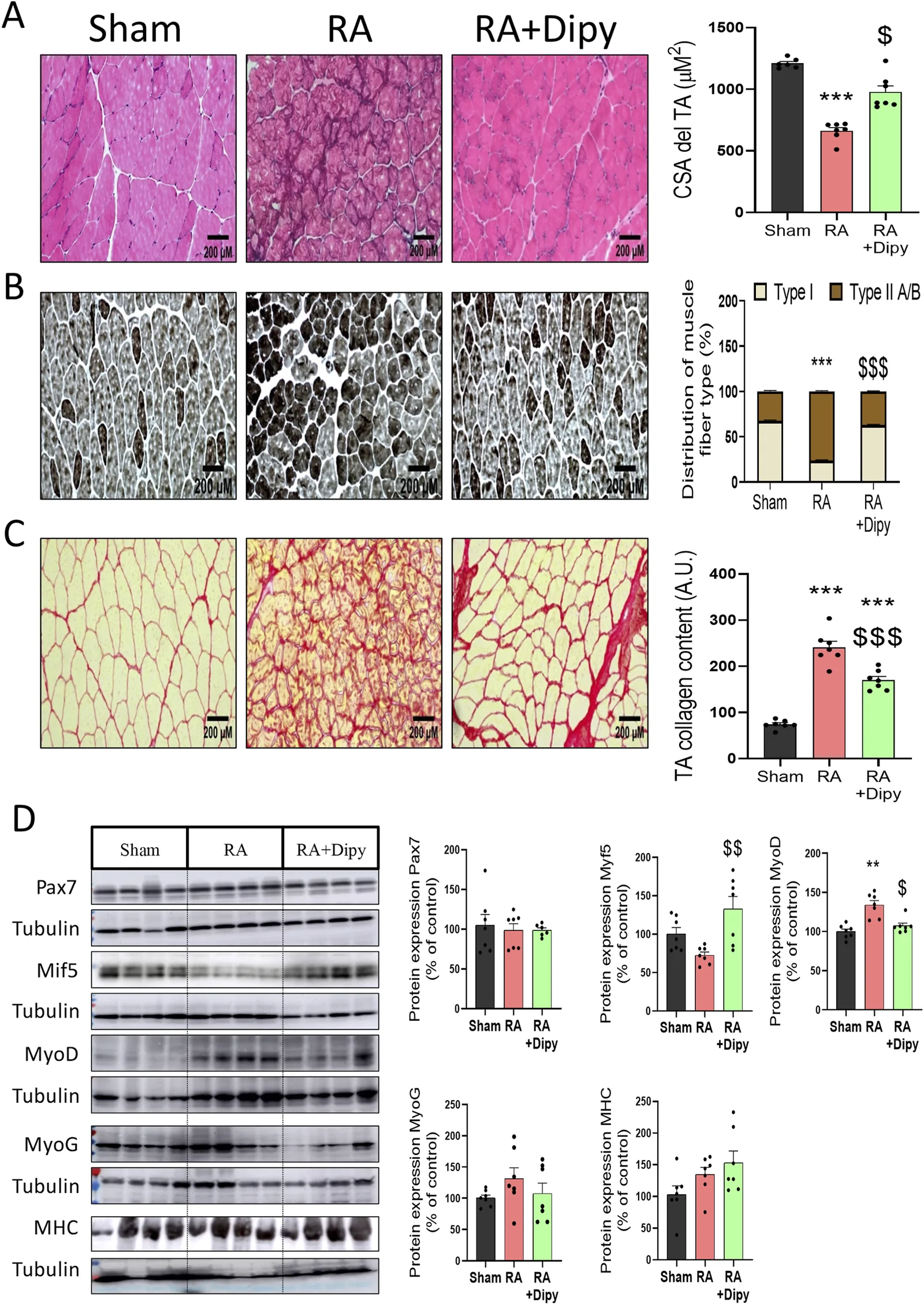
Dipyridamole retrieves the muscle architecture and the myogenic alterations in RA mice after remission. Histology of TA analyzed with **(A)** CSA in H&E staining, **(B)** ATPase staining and **(C)** Collagen content with Picrosirius Red. **(D)** Western blot of Pax7, Myf5, MyoD, MyoG and MHC in the GA of RA model. TA images were acquired at ×20 magnification. Differences are represented as: * Sham vs other groups; $ RA vs. RA + Dipy. Single symbol represents p < 0.05, double symbol represents p < 0.01 and triple symbol represents p < 0.005. Results are presented as mean ± SEM. N = 7 per group. Black arrow indicates the days of K/BxN serum administration. Green arrow indicates the first day of dipyridamole administration in RA mice. RA: Rheumatoid Arthritis; RA + Dipy: Therapeutic treatment with dipyridamole from week 4 after RA induction; CSA: Cross-sectional area; GA: Gastrocnemius; TA: Tibialis anterior; Pax7: Paired box 7; Myf5: Myogenic factor 5; MyoD: Myoblast Determination protein 1; MyoG: Myogenin; MHC: Myosin heavy chain.

Paired-Box 7 (Pax7) expression was unchanged in GA of RA mice with or without dipyridamole. Dipyridamole treatment promotes Myf5 expression higher than those observed in Sham or RA mice. On the other hand, high expression of MyoD was reported in the GA of RA mice, which is suppressed by dipyridamole treatment (Figure 2D). No differences between groups had been reported for myogenin (MyoG) and myosin heavy chain (MHC) expression in GA muscle (Figure 2D).

### 3.3 The anti-catabolic effects of dipyridamole in skeletal muscle are associated with restoration of adenosine levels, A2BR expression and AMPK signaling

Our previous study demonstrated that dipyridamole counteracts muscle atrophy and inflammation through increased muscle adenosine levels. We therefore assessed purinergic and muscle signaling after RA remission.

While inosine levels remained unmodulated across all experimental groups, the RA model was characterized by a disruption of the purinergic system signaling (Figure 3A). Specifically, adenosine and AMP levels were significantly depleted in RA mice compared to healthy controls (Figure 3A). Conversely, ATP levels were elevated in the RA GA muscle, resulting in a drastically reduced AMP/ATP ratio (Figure 3A). Therapeutic administration of dipyridamole successfully reversed these defects, restoring adenosine and AMP concentrations and normalizing the AMP/ATP ratio to levels like those in Sham mice (Figure 3A). Notably, while ADP levels were reduced in RA mice, they were not modulated by dipyridamole (Figure 3A).

**FIGURE 3:**
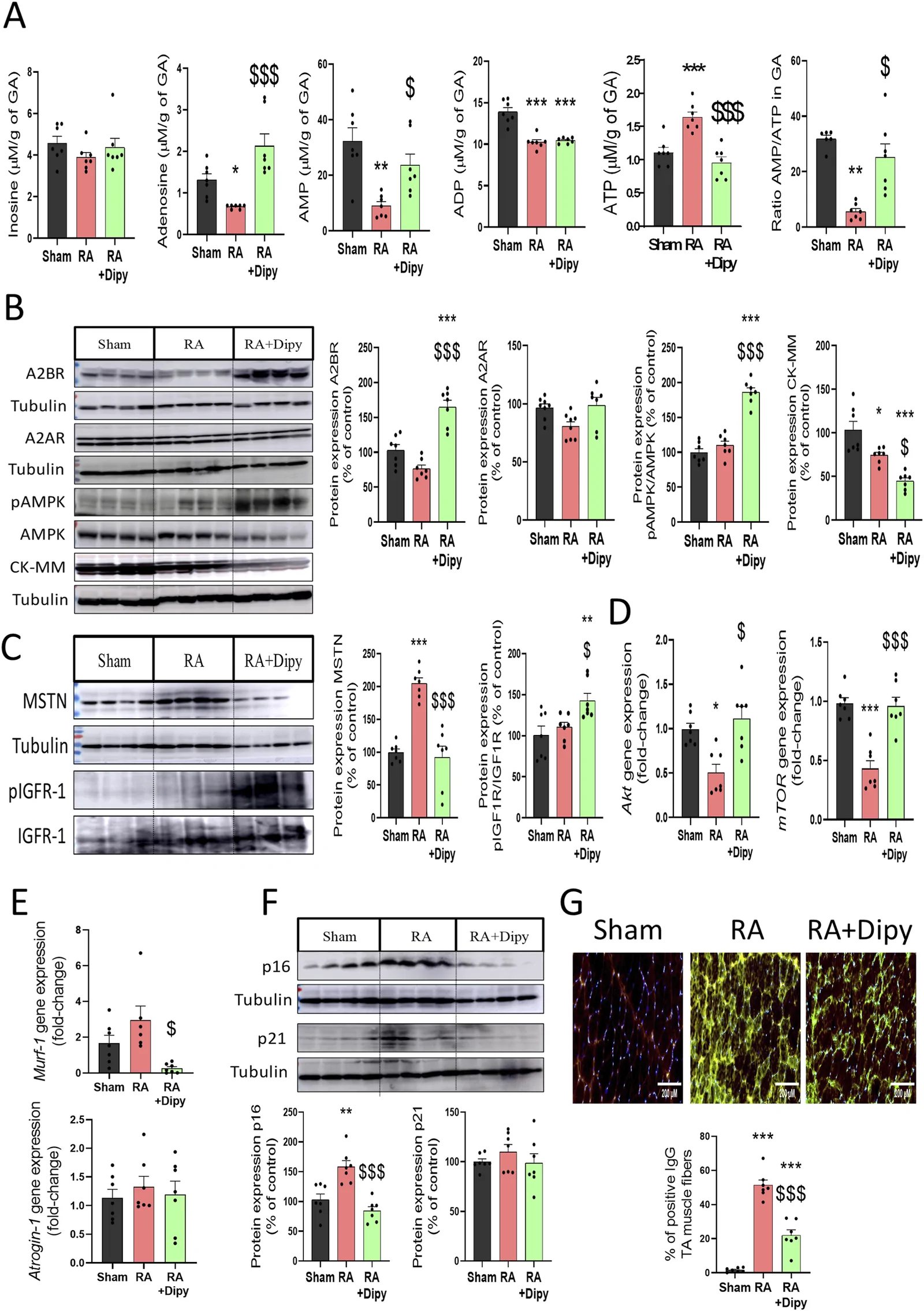
Dipyridamole is associated with activation of the A2BR-AMPK pathway and elevated adenosine and AMP levels, together with stimulation of muscle anabolic processes and reduced cellular senescence and necrotic cell death. **(A)** Nucleoside and nucleotide muscle levels via HPLC in GA. **(B)** A2BR, A2AR, pAMPK/AMPK and CK-MM expression via western blot in GA. **(C)** MSTN and pIGF1R/IGF1R expression via Western blot in GA. **(D)** *Akt, mTOR*, **(E)** *Murf-1* and *Atrogin-1* gene expression via RT-PCR in GA. **(F)** p16 and p21 expression via Western blot in GA. **(G)** Myonecrosis study via immunofluorescence of IgG infiltration in muscle fibers of TA. TA images were acquired at ×20 magnification. Differences are represented as: * Sham vs other groups; $ RA vs. RA + Dipy. Single symbol represents p < 0.05, double symbol represents p < 0.01 and triple symbol represents p < 0.005. Results are presented as mean ± SEM. N = 7 per group. RA: Rheumatoid Arthritis; RA + Dipy: Therapeutic treatment with dipyridamole from week 4 after RA induction; GA: Gastrocnemius; TA: Tibialis anterior; AMP: Adenosine monophosphate; ADP: Adenosine diphosphate; ATP: Adenosine triphosphate; A2BR: Adenosine receptor A2B; A2AR: Adenosine receptor A2A; pAMPK: phospho-AMP-activated protein kinase; CK-MM: Creatine kinase of skeletal muscle; MSTN: Myostatin; pIGF1R: phospho-insulin-like growth factor 1 receptor; *mTOR*: mechanistic target of rapamycin; *Murf-1*: Muscle RING Finger-1; *Atrogin-1*: Muscle atrophy f-box; IgG: immunoglobulin G.

These metabolic shifts directly correlated with the modulation of downstream signaling effectors. Building on our previous findings that dipyridamole counteracts atrophy through increased adenosine, we observed that A2BR expression and phospho-(p)AMPK/AMPK activation were significantly upregulated in dipyridamole-treated RA mice (Figure 3B). In addition, a reduction of creatine kinase of skeletal muscle (CK-MM) expression was found in RA muscle, which was enhanced with dipyridamole treatment in RA mice (Figure 3B).

Consistent with muscle atrophy persistence during inflammatory remission, RA mice exhibited increased MSTN expression in GA muscle (Figure 3C), accompanied by suppression of the *Akt/mTOR* anabolic pathway (Figure 3D). Dipyridamole treatment reduced MSTN and stimulated IGFR-1 expression, promoting *Akt/mTOR* pathway (Figures 3C,D).

Although no differences were observed in the expression of the ubiquitin-proteasome-related genes *Murf-1* or *Atrogin-1* between RA and Sham mice, dipyridamole treatment significantly reduced *Murf-1* expression in the GA muscle of RA mice (Figure 3E).

RA muscle also showed increased expression of the senescence marker p16, which was reversed by dipyridamole treatment. In contrast, p21 expression remained unchanged in RA mice, regardless of dipyridamole administration (Figure 3F).

Histological analysis revealed elevated IgG infiltration within muscle fibers of RA mice, indicative of muscle fiber necrosis. Dipyridamole markedly reduced IgG-positive fibers in RA muscle (Figure 3G), supporting its protective effect against sarcopenic imprint damage in RA.

## 4 Discussion

Previously, we showed that the K/BxN model develops muscle loss associated with systemic and local inflammation, and that dipyridamole reduced disease activity while exerting anabolic effects on muscle via increased adenosine availability and stimulation of A2AR and A2BR (Marco-Bonilla et al., 2025). Here, we demonstrate that a persistent sarcopenic imprint persists following both systemic and joint swelling remission in experimental RA, as observed in many patients receiving current therapies (Hein et al., 2022; Hasegawa et al., 2023; Kulyk and Dzhus, 2025). This effect, observed after remission of joint swelling, suggests therapeutic potential for prevalent sarcopenia in RA remission.

These findings show that muscle pathology persists despite inflammatory control (Hasegawa et al., 2023). Although clinical strategies focus on achieving remission with MTX and biologic DMARDs, muscle homeostasis is not restored (Hasegawa et al., 2023; Kulyk and Dzhus, 2025). In the K/BxN serum-transfer model we reported loss of TA and GA mass, WAT gain, fiber atrophy, fibrosis, and impaired grip strength and endurance persisted despite remission of joint swelling. Dipyridamole treatment reverses this persistent muscle imprint by restoring adenosine-mediated signaling, possibly via the A2BR receptor in the muscle. It also may promote an anabolic response associated with IGF1R activation and MSTN suppression*.* Thus, RA-associated sarcopenia persists despite resolution of joint swelling and clinical remission. It has been previously described that muscle atrophy and weakness are already present before pain and swelling affect the physical activity of the RA patient, indicating that muscle is affected from early stage disease (Costamagna et al., 2015).

In contrast to the BAT loss and reduced rotarod performance observed during peak inflammation (Marco-Bonilla et al., 2025), both parameters were normalized after remission, indicating that some systemic features may depend directly on inflammatory signaling (Moon et al., 2020).

While we previously reported inflammation-induced muscle proliferation (Marco-Bonilla et al., 2025), this response was absent after remission. Instead, MyoD was selectively upregulated, consistent with impaired regeneration and satellite cell exhaustion (Asakura et al., 2007). The sarcopenic imprint was characterized by sustained MSTN elevation, *Akt*/*mTOR* suppression, and failure of A2BR/AMPK signaling (Gnad et al., 2020; Marco-Bonilla et al., 2023; Marco-Bonilla et al., 2025), supporting a persistent catabolic and metabolic reprogramming.

After remission, dipyridamole restored adenosine availability, reactivated A2BR/AMPK signaling, reduced MSTN, and stimulated IGF1R, thereby recovering muscle anabolic capacity, consistent with reports in aging sarcopenia (Gnad et al., 2020). Unlike during acute inflammation, when A2AR activation limited its antifibrotic effect (Marco-Bonilla et al., 2025), dipyridamole reduced fibrosis after remission, suggesting a context-dependent role of A2AR in modulating inflammation and collagen deposition (Zhang et al., 2025). The reduction of CK expression following dipyridamole treatment in RA muscle is linked to the necessary adjustment of ATP levels. High ATP levels are associated with the fast-switch Type II fibers seen in RA mice. By lowering these levels, dipyridamole helps the muscle shift back toward the slow-twitch Type I fibers found in Sham mice (Talbot and Maves, 2016).

The impairment of muscle regeneration in RA muscle is supported by the upregulation of the apoptotic mediator p16 and the pronounced myofiber necrosis observed. Notably, no increase in muscle atrophy markers was detected, consistent with reduced atrophic signaling at later stages of muscle fibers degeneration (Wan et al., 2021). In contrast, the improvement of muscle anabolism by dipyridamole in RA muscle blocked the progression to senescence and necrosis.

Together, these findings demonstrate that RA leaves a persistent sarcopenic imprint that remains after clinical RA resolution. This sarcopenic imprint is characterized by impaired regeneration, metabolic reprogramming, fibrosis, and sustained catabolic signaling despite clinical remission. By restoring adenosine availability and reactivating A2BR/AMPK signaling, dipyridamole reverses this sarcopenia through MSTN suppression and IGF1R-dependent anabolic recovery, while reducing muscle fibrosis. These results redefine RA-associated sarcopenia as a prevalent comorbidity that persists despite inflammatory control and identify modulation of adenosine signaling, potentially involving A2BR, as a promising therapeutic strategy to restore muscle integrity and function in RA patients in remission. In humans, this persistent muscle deficit may contribute to the development of frailty syndrome at older ages, highlighting the importance of early therapeutic intervention during active diseases.

Several limitations of the present study should be acknowledged. First, although dipyridamole treatment was associated with restoration of adenosine availability, increased A2BR expression, AMPK activation, and improved muscle anabolic signaling, the study did not include A2BR loss-of-function approaches, such as receptor antagonism or genetic deletion. Therefore, a causal dependency on A2BR signaling cannot be definitively established, and the observed associations should be interpreted as correlative. In addition, dipyridamole is not a selective A2BR modulator and may affect multiple components of the purinergic system, which could also contribute to the observed therapeutic effects. Second, although treatment was initiated after the remission time point previously characterized in this model, inflammatory mediators were not directly quantified in the current cohort at study completion. Consequently, the reported effects should be interpreted as occurring in a post-remission setting rather than being definitively independent of residual inflammation. Third, the study did not include a conventional DMARD or anti-inflammatory comparator group, which would have helped distinguish the effects associated with resolution of inflammation from direct muscle-specific effects of dipyridamole. Finally, the study was conducted in a relatively small cohort of male mice and lacks validation in female animals and human patients. Therefore, further studies incorporating larger cohorts, both sexes, mechanistic A2BR-targeted approaches, direct inflammatory assessments, and clinical validation will be required to confirm the translational relevance of these findings.

## Data Availability

The raw data supporting the conclusions of this article will be made available by the authors, without undue reservation.

## References

[B1] Aartsma-Rus A. Van Putten M. (2014). Assessing functional performance in the mdx mouse model. JoVE 51303. 10.3791/51303-v PMC4158772 24747372

[B2] Allahham M. Lerman A. Atar D. Birnbaum Y. (2022). Why not dipyridamole: a review of current guidelines and Re-evaluation of utility in the modern era. Cardiovasc Drugs Ther. 36, 525–532. 10.1007/s10557-021-07224-9 34245446 PMC8271326

[B3] Asakura A. Hirai H. Kablar B. Morita S. Ishibashi J. Piras B. A. (2007). Increased survival of muscle stem cells lacking the MyoD gene after transplantation into regenerating skeletal muscle. Proc. Natl. Acad. Sci. U. S. A. 104, 16552–16557. 10.1073/pnas.0708145104 17940048 PMC2034220

[B4] Bennett J. L. Pratt A. G. Dodds R. Sayer A. A. Isaacs J. D. (2023). Rheumatoid sarcopenia: loss of skeletal muscle strength and mass in rheumatoid arthritis. Nat. Rev. Rheumatol. 19, 239–251. 10.1038/s41584-023-00921-9 36801919

[B5] Costamagna D. Costelli P. Sampaolesi M. Penna F. (2015). Role of inflammation in muscle homeostasis and myogenesis. Mediat. Inflamm. 2015, 805172. 10.1155/2015/805172 26508819 PMC4609834

[B6] Giles J. T. Ling S. M. Ferrucci L. Bartlett S. J. Andersen R. E. Towns M. (2008). Abnormal body composition phenotypes in older rheumatoid arthritis patients: association with disease characteristics and pharmacotherapies. Arthritis and Rheumatism 59, 807–815. 10.1002/art.23719 18512711 PMC2670994

[B7] Gnad T. Navarro G. Lahesmaa M. Reverte-Salisa L. Copperi F. Cordomi A. (2020). Adenosine/A2B receptor signaling ameliorates the effects of aging and counteracts obesity. Cell Metab. 32, 56–70.e7. 10.1016/j.cmet.2020.06.006 32589947 PMC7437516

[B8] Hasegawa E. Ito S. Kurosawa Y. Kobayashi D. Otani H. Abe A. (2023). The efficacy of biological disease-modifying antirheumatic drugs on sarcopenia in patients with rheumatoid arthritis. Intern. Med. 62, 373–379. 10.2169/internalmedicine.9600-22 36725065 PMC9970816

[B9] Hein T. R. Peterson L. Bartikoski B. J. Portes J. Espírito Santo R. C. Xavier R. M. (2022). The effect of disease-modifying anti-rheumatic drugs on skeletal muscle mass in rheumatoid arthritis patients: a systematic review with meta-analysis. Arthritis Res. Ther. 24, 171. 10.1186/s13075-022-02858-y 35854372 PMC9295282

[B10] Kulyk M. Dzhus M. (2025). Assessment of sarcopenia in young patients with inflammatory arthritis: a cross-sectional study. Sci. Rep. 15, 8236. 10.1038/s41598-025-88939-9 40064969 PMC11893876

[B11] Marco-Bonilla M. Herencia R. Fresnadillo M. Huete-Toral F. Carracedo G. Largo R. (2023). Dipyridamole activates adenosine A2B receptor and AMPK/cAMP signaling and promotes myogenic differentiation of myoblastic C2C12 cells. Front. Pharmacol. 14, 1247664. 10.3389/fphar.2023.1247664 37771723 PMC10522837

[B12] Marco-Bonilla M. Fresnadillo M. Sanchez-Platero I. de la Riva-Bueno M. Huete-Toral F. Carracedo G. (2025). Dual action of dipyridamole in experimental rheumatoid arthritis: suppression of joint inflammation and upregulation of muscle anabolism via adenosine and AMPK pathways. Arthritis and Rheumatology n/a 78, 1535–1553. 10.1002/art.70047 41466573 PMC13313103

[B13] Moon J. Kim D. Kim E. K. Lee S. Na H. S. Kim G. N. (2020). Brown adipose tissue ameliorates autoimmune arthritis via inhibition of Th17 cells. Sci. Rep. 10, 12374. 10.1038/s41598-020-68749-x 32704024 PMC7378076

[B14] Moschou D. Krikelis M. Georgakopoulos C. Mole E. Chronopoulos E. Tournis S. (2023). Sarcopenia in rheumatoid arthritis. A narrative review. JFSF 8, 44–52. 10.22540/JFSF-08-044 36873824 PMC9975974

[B15] Ohta A. Sitkovsky M. (2014). Extracellular adenosine-mediated modulation of regulatory T cells. Front. Immunol. 5, 304. 10.3389/fimmu.2014.00304 25071765 PMC4091046

[B16] Pinzi L. Bisi N. Rastelli G. (2024). How drug repurposing can advance drug discovery: challenges and opportunities. Front. Drug Discov. 4, 1460100. 10.3389/fddsv.2024.1460100

[B17] Talbot J. Maves L. (2016). Skeletal muscle fiber type: using insights from muscle developmental biology to dissect targets for susceptibility and resistance to muscle disease. Wiley Interdiscip. Rev. Dev. Biol. 5, 518–534. 10.1002/wdev.230 27199166 PMC5180455

[B18] Vincenzi F. Pasquini S. Contri C. Cappello M. Nigro M. Travagli A. (2023). Pharmacology of adenosine receptors: recent advancements. Biomolecules 13, 1387. 10.3390/biom13091387 37759787 PMC10527030

[B19] Wan M. Gray-Gaillard E. F. Elisseeff J. H. (2021). Cellular senescence in musculoskeletal homeostasis, diseases, and regeneration. Bone Res. 9, 41. 10.1038/s41413-021-00164-y 34508069 PMC8433460

[B20] Zhang X. Sun J. Guo J. Hu X. Zhou J. Xie X. (2025). Inhibition of adenosine/A2A receptor signaling suppresses dermal fibrosis by enhancing fatty acid oxidation. Cell Commun. Signal 23, 206. 10.1186/s12964-025-02210-2 40301970 PMC12042438

